# IMPLEMENTATION CHALLENGES AND OPPORTUNITIES IN A COMMUNITY-BASED TRANSITIONAL CARE SERVICES PROGRAM

**DOI:** 10.1093/geroni/igz038.1855

**Published:** 2019-11-08

**Authors:** Raven H Weaver, Cory Bolkan

**Affiliations:** 1 Washington State University, Pullman, Washington, United States; 2 Washington State University, Vancouver, Washington, United States

## Abstract

High-risk older adults (i.e., low-income, chronically ill) often have complex, costly healthcare needs and are at risk of re-hospitalization. Hospitals traditionally lead efforts to reduce readmissions, while community-based aging services organizations (e.g., Area Agency on Aging AAA) offer older adults in-home health, social support, and information/referral to community resources. Thus, creating, sustaining, and scaling up hospital-community partnerships can better meet older adults’ comprehensive needs. We evaluated efforts of a hospital-AAA project to develop and implement a local transitional care services program (TCSP) that provided in-home/phone support post-discharge for high-needs older adults. Over a four-year period, 1,921 individuals (mean= 75 years; 57% women) were referred from hospital as eligible for TCSP. After referral, however, only 22.8% were successfully connected to community-based services and men were more likely than women to complete TCSP (Χ2= 6.92; p= .009). Of those referred, only 4% were re-hospitalized, indicating potential success of TCSP. Data revealed most were unable to be contacted (27.9%), refused the program (21.6%) or utilized alternative services, including SNFs (20.3%); inconsistent data collection and procedures yielded problematic missing data and inability to assess reasons for low engagement. We also surveyed and interviewed AAA staff (n=16) and found most staff exhibited high readiness for evidence-informed practices, supported proactive data use to improve planning, advocating, and serving clients, and identified significance of multidisciplinary community partnerships. Our findings generated recommendations to enhance staff engagement in TCSP, improve data collection for transforming data utility beyond enrollment purposes, and consider programmatic modifications to reach vulnerable elders.

